# L‐Se‐methylselenocysteine sensitizes lung carcinoma to chemotherapy

**DOI:** 10.1111/cpr.13038

**Published:** 2021-04-01

**Authors:** Jia Ma, Jing Huang, Jinli Sun, Yanfeng Zhou, Xiaoyuan Ji, Daoxia Guo, Chang Liu, Jiyu Li, Jiye Zhang, Haiyun Song

**Affiliations:** ^1^ School of Pharmacy Health Science Center Xi'an Jiaotong University Xi'an China; ^2^ State Key Laboratory of Oncogenes and Related Genes Center for Single‐Cell Omics School of Public Health Shanghai Jiao Tong University School of Medicine Shanghai China; ^3^ Department of Neurology Xuhui District Central Hospital Shanghai China; ^4^ Henan Xibaikang Health Industry Co., Ltd Jiyuan China

**Keywords:** chemotherapy, lipid peroxidation, lung carcinoma, methylselenocysteine

## Abstract

**Objectives:**

Organic Selenium (Se) compounds such as L‐Se‐methylselenocysteine (L‐SeMC/SeMC) have been employed as a class of anti‐oxidant to protect normal tissues and organs from chemotherapy‐induced systemic toxicity. However, their comprehensive effects on cancer cell proliferation and tumour progression remain elusive.

**Materials and Methods:**

CCK‐8 assays were conducted to determine the viabilities of cancer cells after exposure to SeMC, chemotherapeutics or combined treatment. Intracellular reactive oxygen species (ROS) levels and lipid peroxidation levels were assessed via fluorescence staining. The efficacy of free drugs or drug‐loaded hydrogel against tumour growth was evaluated in a xenograft mouse model.

**Results:**

Among tested cancer cells and normal cells, the A549 lung adenocarcinoma cells showed higher sensitivity to SeMC exposure. In addition, combined treatments with several types of chemotherapeutics induced synergistic lethality. SeMC promoted lipid peroxidation in A549 cells and thereby increased ROS generation. Significantly, the in vivo efficacy of combination therapy was largely potentiated by hydrogel‐mediate drug delivery.

**Conclusions:**

Our study reveals the selectivity of SeMC in the inhibition of cancer cell proliferation and develops an efficient strategy for local combination therapy.

## Graphical abstract

L‐Se‐Methylselenocysteine (SeMC) induces lipid peroxidation to suppress the proliferation of lung cancer cells and sensitizes them to a variety of chemotherapeutic agents. Hydrogel‐mediated combination therapy with SeMC and chemotherapeutics achieves markedly enhanced efficacy on the inhibition of tumour growth comparing to conventional administration.

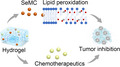

## 1 INTRODUCTION

Chemotherapy is one of the most broadly used and effective ways for cancer treatment. In general, chemotherapeutic drugs inhibit cancer cell growth via the induction of cell cycle arrest and/or oxidative damage‐triggered apoptosis.^1^, ^2^, ^3^, ^4^, ^5^ In the meantime, these agents have unavoidable side effects on normal tissues and organs. Therefore, cytoprotective agents are desirable during chemotherapy. Low doses of Se have been found to promote anti‐oxidant activity, protecting membrane lipids and macromolecules from peroxides‐mediated oxidative damage.^6^, ^7^, ^8^ Among the Se compounds, the clinical application of inorganic Se compounds is limited due to their high water solubility, poor liposolubility, and high mutagenic and genotoxic properties. On the contrary, organic Se compounds such as SeMC, selenocystine and selenomethionine pass the cell membrane more efficiently and exhibit fewer side effects and lower systemic toxicity, thus holding great potential in cancer therapy.^9^, ^10^, ^11^

Low levels of Se metabolites can incorporate into and form active sites of a number of Se‐containing proteins including glutathione peroxidases (GPX), glutathione reductase (GR) and thioredoxin reductases (TrxR), which function as enzymes to regulate intracellular redox status and prevent oxidative damage from exogenous stimuli.^12^, ^13^, ^14^, ^15^, ^16^ In contrast, medium‐to‐high doses of Se compounds lead to increased production of hydrogen selenide (HSe^‐^) and methyl selenol (CH3Se^‐^), which act as pro‐oxidants to interfere with intracellular redox balance and induce the formation of superoxide and hydrogen peroxide.^17^, ^18^ Consequently, increased ROS generation may render cancer cells more susceptible to chemotherapeutic agents.^19^, ^20^, ^21^, ^22^ Therefore, it is reasonable to apply medium‐to‐high doses of Se compounds, lower the threshold of cancer cells on ROS tolerance and enhance the efficacy of chemotherapy.

Conventional drug administration depends on blood circulation and is apt to cause systemic effect on normal tissues and organs. Besides, the lack of targeted delivery results in low bioavailability of drugs in the tumour tissue with short retention time.^23^ In comparison, hydrogel‐based drug delivery platform represents an intelligent strategy to address these issues.^24^, ^25^ Particularly, in situ formation of hydrogel allows local drug delivery, increases drug concentration at tumour foci and reduces systemic exposure. The design of tumour microenvironment (TME)‐responsive hydrogel scaffolds enables tunable hydrogel disassembly, providing continuous and controllable release of therapeutic agents.^26^, ^27^, ^28^, ^29^, ^30^

Herein, we examined the effects of SeMC on the viabilities of several types of cancer and non‐cancer cells and found that the A549 lung adenocarcinoma cell line was more sensitive to SeMC treatment than other cell lines. Combining SeMC with chemotherapeutics such as Epirubicin (EBN), 5‐Fluorouracil (5‐Fu), Gemcitabine (GEM), Cisplatin (CDDP) or Paclitaxel (PTX) produced synergistic effects on A549 cell death. We further showed that SeMC‐induced lipid peroxidation to increase the ROS levels in A549 cells and sensitized them to chemotherapeutic agents. Lastly, we utilized a type of TME‐responsive hydrogel for combined local delivery of SeMC and EBN to A549 tumours in a mouse xenograft model and achieved markedly enhanced efficacy on the inhibition of tumour growth comparing to conventional administration.

## 2 MATERIALS AND METHODS

### 2.1 Cell culture and reagents

293T embryonic kidney cells, L6 myoblast cells, A549 lung carcinoma cells, CT26 colon carcinoma cells, 4T1 mammary carcinoma cells and Hepa1‐6 hepatoma cells were obtained from the Cell Bank of Chinese Academy of Sciences (Shanghai). The cells were cultured in a humidified incubator at 37°C with 5% CO_2_. 293T cells, L6 cells and Hepa1‐6 cells were cultured in Dulbecco's modified Eagle medium (DMEM, Gibco). A549 cells, CT26 cells and 4T1 cells were maintained in RPMI 1640 medium (Gibco). All media were supplemented with 10% foetal bovine serum (FBS, Gibco), 100 U/mL streptomycin (Invitrogen) and 100 U/mL penicillin (Invitrogen). SeMC was from Henan Xibaikang Health Industry Co., Ltd. EBN, GEM, PTX, CDDP and 5‐Fu were from MedChemExpress. Cell counting kit‐8 (CCK‐8) was from Beyotime. 2’,7’‐dichlorofluorescin diacetate (DCFH‐DA) was from Sigma‐Aldrich (D6883). BODIPY C11 was from Molecular Probes (D3861).

### 2.2 Cell viability assay

Cells were seeded in 96‐well plates at a density of 5 × 10^3^ cells per well and cultured for 12 hours at 37°C. After incubation with 50‐200 μM SeMC (9.1‐36.4 μg/mL), 0.2 μg/mL EBN, 1 μg/mL GEM, 10 ng/mL PTX, 3 μg/mL CDDP or 2.5 μg/mL 5‐Fu for 24‐48 hours, the cells were incubated with fresh media containing 10% CCK‐8 solution for 30 minutes at 37°C. The viabilities of the cells were determined by measuring the absorbance at 450 nm with a microplate reader.

### 2.3 Preparation and characterization of hydrogel

The ROS‐responsive linker *N^1^*‐(4‐boronobenzyl)‐*N^3^*‐(4‐boronophenyl)‐*N^1^,N^1^,N^3^,N^3^*‐tetramethylpropane‐1,3‐diaminium (TSPBA) was prepared as follows: First, *N^1^,N^1^,N^3^,N^3^*‐tetramethylpropane‐1,3‐diaminium (Sigma‐Aldrich) and 4‐(bromomethyl)‐phenylboronic acid (Sigma‐Aldrich) were added to dimethylformamide (Sigma‐Aldrich) and dissolved thoroughly. After stirring at 60°C overnight, the mixture was washed with tetrahydrofuran (Sigma‐Aldrich). Afterwards, the mixture was filtered and dried. The poly(vinylalcohol) (PVA, 72 kDa, Aladdin) matrix was dissolved in deionized water. The solution was stirred for 12 hours at 90°C. The hydrogel was formed by mixing the TSPBA linker with the PVA matrix. The Cryo‐SEM (SU8010, Hitachi) was utilized to characterize the surface morphology of the hydrogel.

To measure the kinetics of hydrogel disassembly and drug release, EBN was mixed with the PVA matrix before the addition of the TSPBA linker for the formation of EBN@Gel. The gel‐to‐sol transition upon H_2_O_2_ treatment was monitored at different time points by determining the amount of EBN in the produced supernatant with the UV‐Vis‐NIR absorbance spectrum (SHIMADZU, UV1800).

### 2.4 Detection of ROS and lipid peroxides

A549 cells (4 × 10^5^ cells per well) were cultured in 24‐well plates overnight. After incubation with SeMC and/or therapeutic drugs for 12 hours, the cells were washed twice with PBS and incubated with DMEM containing 20 μM DCFH‐DA for 45 minutes. Fluorescence imaging was performed on a Leica TCS SP8 confocal laser scanning microscope (Ex = 488 nm, Em = 520 nm). For detection of intracellular lipid peroxides levels, the cells were stained with 2 μM BODIPY C11 in DMEM for 60 minutes after drug treatment and PBS wash and were imaged by the confocal laser scanning microscope (Ex = 500/581 nm, Em = 510/591 nm).

### 2.5 Animal treatment

BALB/c nude mice (female, 6‐7 weeks old) were purchased from SLAC Laboratory Animal Co. Ltd. All mouse experiments were conducted following protocols approved by the Animal Care and Use Committee of Shanghai Institute of Nutrition and Health, Chinese Academy of Sciences. For tumour cell inoculation, 6 × 10^6^ A549 cells stably expressing luciferase were injected subcutaneously in the mice received on the right flank. When the tumour size reached ~90 mm^3^, the mice were randomly divided into three groups (n = 7 per group) and intratumorally injected with unloaded hydrogel (Gel), free SeMC/EBN (2 mg/kg SeMC and 2 mg/kg EBN) or SeMC/EBN‐loaded hydrogel (SeMC/EBN@Gel, 2 mg/kg SeMC and 2 mg/kg EBN) every four days. For hydrogel administration, 100 μL PVA (7.5 wt%) matrix in the absence or presence of drugs was mixed with 100 μL TSPBA (5 wt%) linker in situ. The tumours were measured by a digital calliper and the tutor size was calculated according to the following formula: (length × width^2^)/2. In addition, in vivo bioluminescence imaging was carried out to monitor tutor progression. The mice were anesthetized with isoflurane, injected intraperitoneally with D‐Luciferin (150 μg/g) and imaged by an IVIS Spectrum Imaging System (Perkin Elmer).

### 2.6 Statistical analysis

Statistical analyses were performed with the GraphPad Prism. All data were representative of >3 independent experiments and presented as mean ±SD or mean ±SEM. Student's *t* test was used to determine the statistical significance of the differences between two groups. ns means not significant, *P < .05, **P < .01, ***P < .001.

## 3 RESULTS

### 3.1 SeMC inhibits A549 cell growth

**FIGURE 1:**
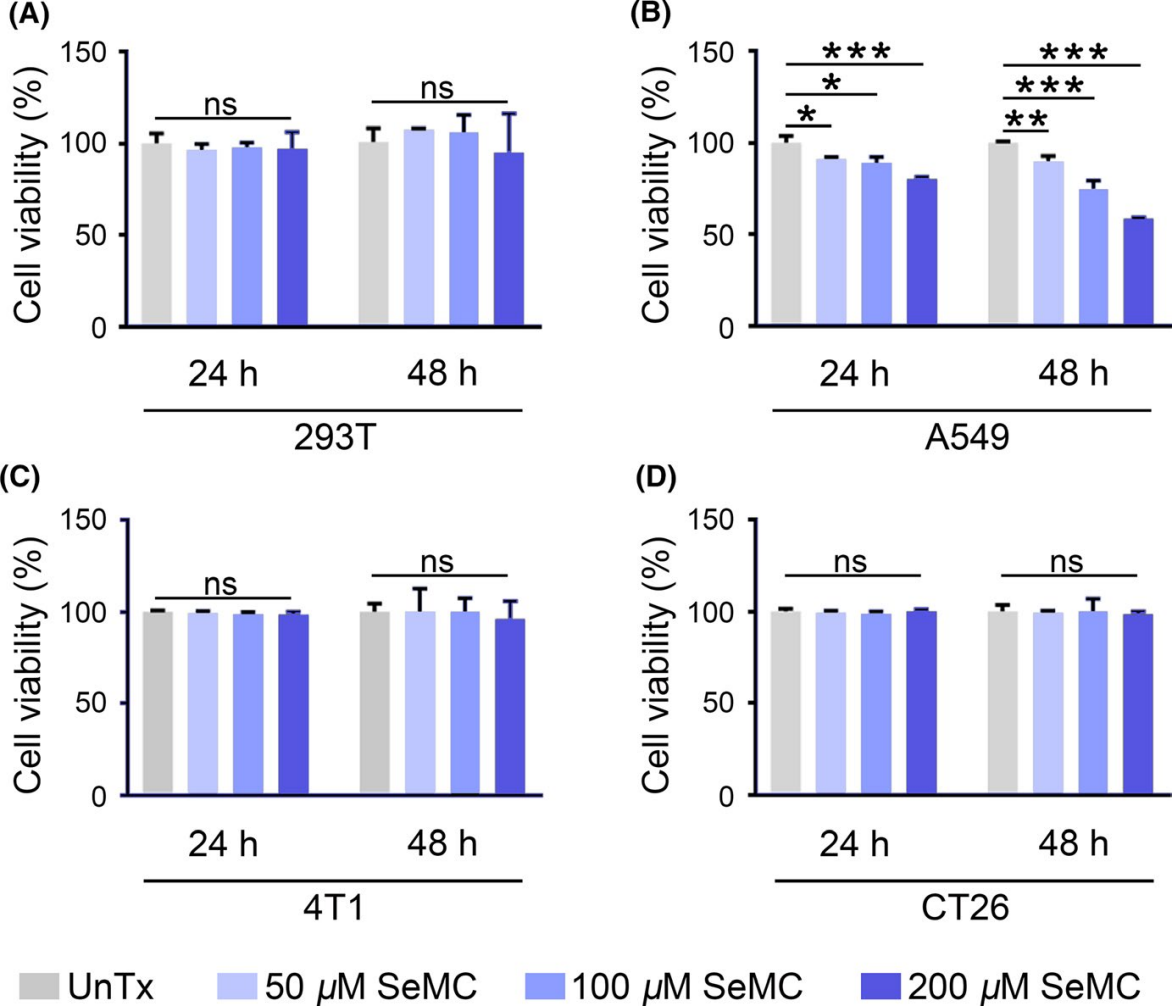
SeMC inhibits the viability of A549 cells. (A‐D) Cytotoxicity assays for 293T cells (A), A549 cells (B), 4T1 cells (C) and CT26 cells (D) in the presence of SeMC for 24 or 48 hours. Data are represented as mean ±SD (n = 3). *P < .05, **P < .01, ***P < .001, ns means not significant

Firstly, we measured the effect of SeMC on the viability of 293T cells and L6 cells, two representative non‐cancer cell lines. At concentrations ranging from 50 to 200 μM, no sign of cytotoxicity was observed (Figure 1A and Figure S1A). Therefore, we tested several cancer cell lines with the same doses of SeMC. A mild reduction in the viability was observed in A549 cells upon exposure to 50 μM SeMC, and this inhibition was aggravated in a dose‐dependent manner (Figure 1B). In contrast, the viabilities of other cancer cell lines including 4T1 mammary carcinoma cells, CT26 colon carcinoma cells and Hepa1‐6 hepatoma cells were not sensitive to the treatment by 50‐200 μM SeMC (Figure 1C,D and Figure S1B). These results suggested that A549 cells were less tolerant to SeMC treatment than other tested cancer and normal cells. We thus focussed on this cell line for further study.

### 3.2 SeMC acts synergistically with chemotherapeutic agents

**FIGURE 2:**
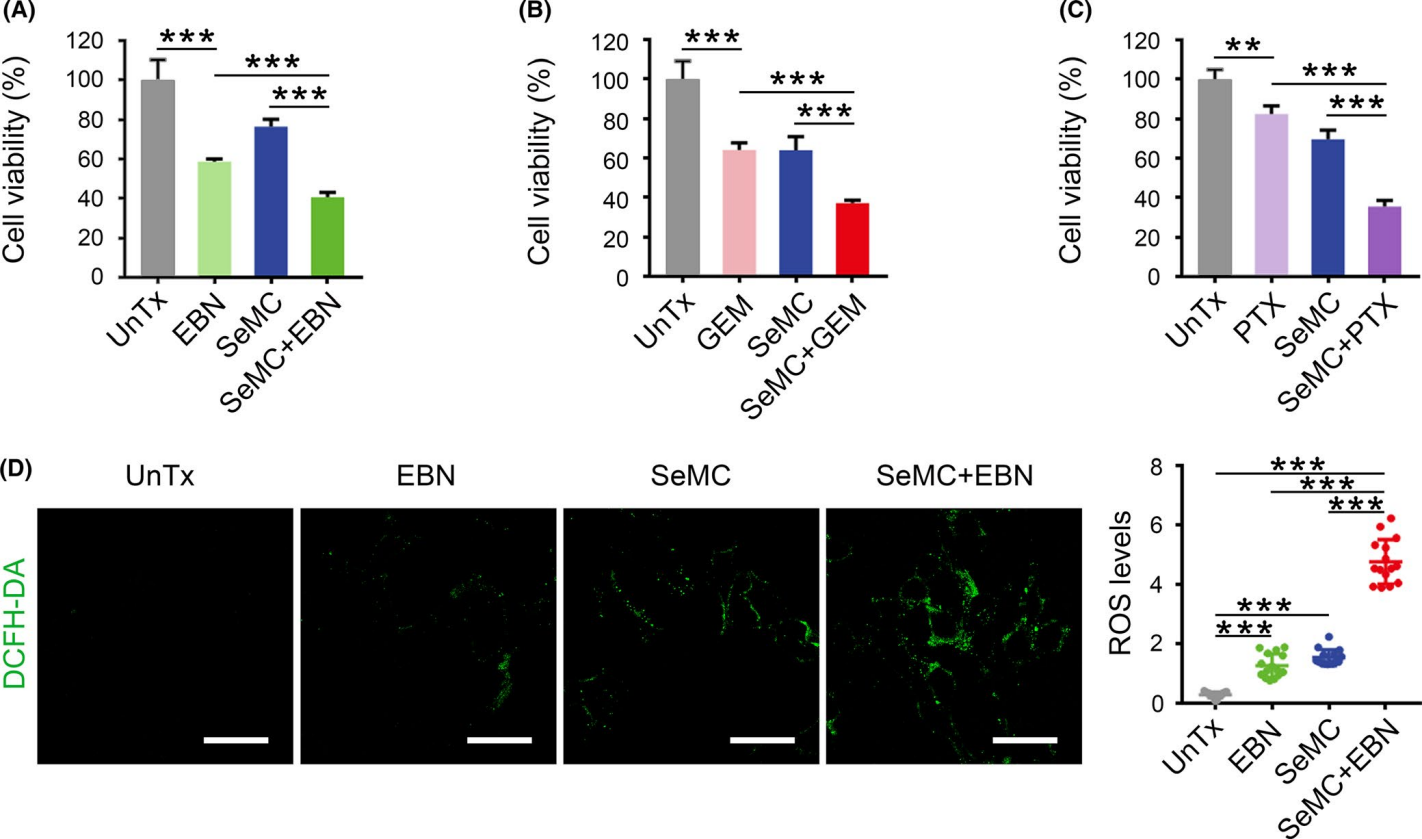
SeMC acts synergistically with chemotherapeutic agents. (A‐C) The viabilities of A549 cells after exposure to 200 μM SeMC, 0.2 μg/mL EBN, 1 μg/mL GEM and 10 ng/mL PTX or their combination for 48 hours. Data are expressed as mean ±SD (n = 3). (D) Fluorescent images and quantification of ROS levels in A549 cells after indicated treatments. Complete data are provided in Figure S2. Scale bars: 50 μm. Data are represented as mean ±SD (n = 15). **P < .01, ***P < .001

Next, we tested the notion that whether the inhibitory effect of SeMC on A549 cell proliferation could improve the efficacy of chemotherapeutic agents. We chose five therapeutics including EBN, 5‐Fu, GEM, CDDP and PTX, which represented first‐line anti‐cancer drugs. While each therapeutic agent could independently inhibit the proliferation of A549 cells, the combination with SeMC noticeably enhanced their activities (Figure 2A‐C and Figure S2A,B). In contrast, treatment with SeMC did not enhance the efficacy of EBN or GEM on 4T1 cells, which further suggested the selectivity of SeMC on A549 cells (Figure S3). The generation of oxidative stress is a common feature for chemotherapeutic agents. It was possible that SeMC treatment increased the basal levels of oxidative stress in A549 cell and thereby rendered them more susceptible to ROS generation by therapeutics. To test this hypothesis, we monitored intracellular ROS levels of A549 cells upon individual or combined SeMC and EBN treatment. Indeed, either SeMC or EBN could individually induce moderate levels of ROS in A549 cells, and their combination strongly increased ROS generation (Figure 2D and Figure S2C).

### 3.3 SeMC induces lipid peroxidation

**FIGURE 3:**
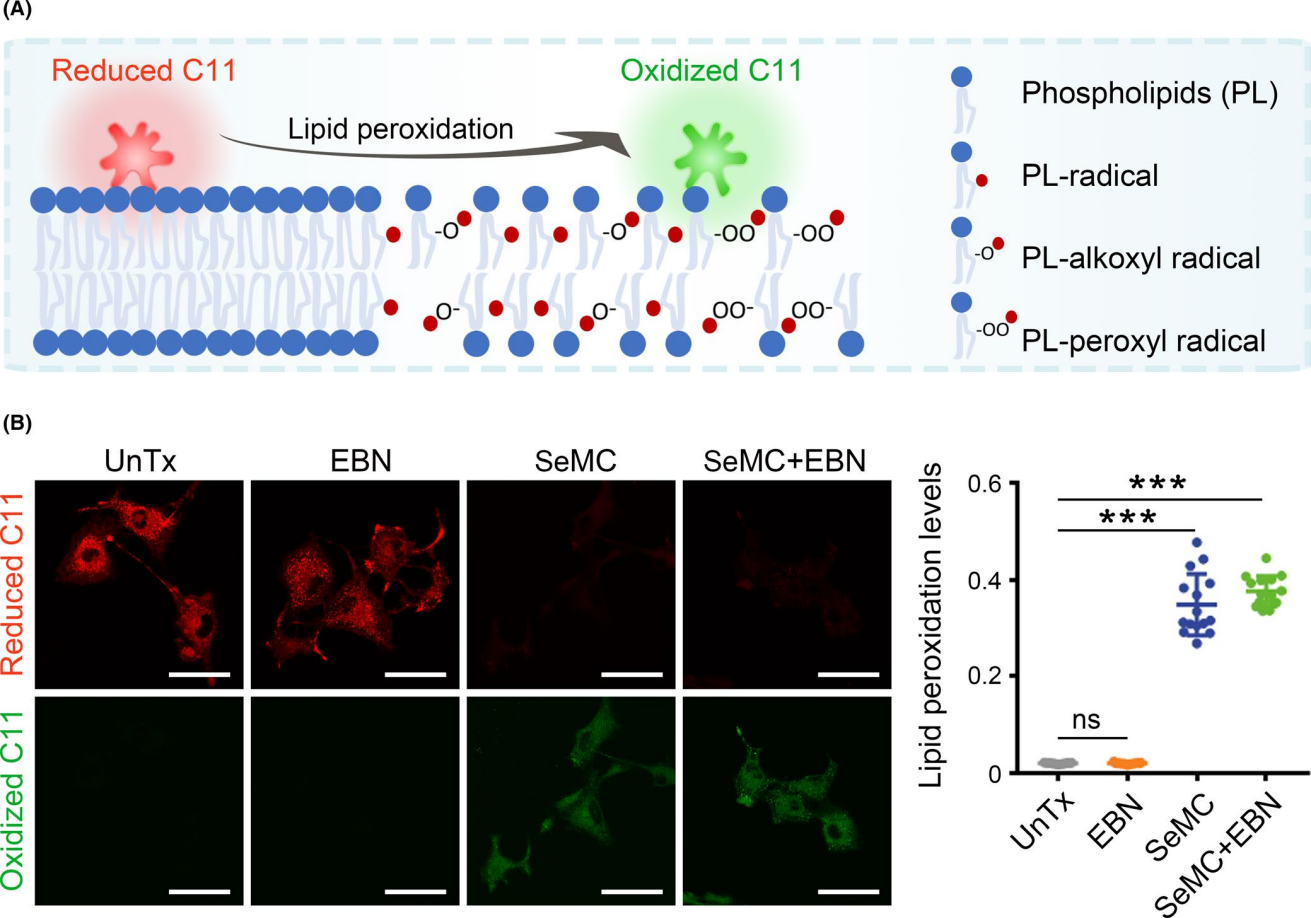
SeMC induces lipid peroxidation. (A) A diagram for the detection of lipid peroxides by BODIPY C11. (B) Left panels: fluorescent images showing reduced form (red) and oxidized form (green) of BODIPY C11 in A549 cells after exposure to 200 μM SeMC, 0.2 μg/mL EBN, or their combination. Scale bars: 50 μm. Right panel: Relative lipid peroxidation levels represented as the ratio of oxidized to reduce BODIPY‐C11. Data are represented as mean ±SD (n = 15). ***P < .001, ns means not significant

Next, we investigated the mechanism under SeMC‐induced ROS generation. Although low levels of Se compounds may assist intracellular redox balance, redundant Se exposure can cause adverse effect via the induction of lipid peroxidation. We determined the levels of lipid peroxidation in A549 cells with BODIPY C11, whose fluorescence signal would shift from red to green upon oxidation (Figure 3A). Individual EBN treatment did not increase the levels of lipid peroxidation in A549 cells. In contrast, incubation with SeMC significantly induced lipid peroxidation, and combined treatment produced an effect similar to that by SeMC alone (Figure 3B). These data indicated that SeMC disturbed intracellular redox homeostasis independent of therapeutic drugs, providing an alternative route for ROS accumulation.

### 3.4 Preparation and characterization of ROS‐responsive hydrogel

**FIGURE 4:**
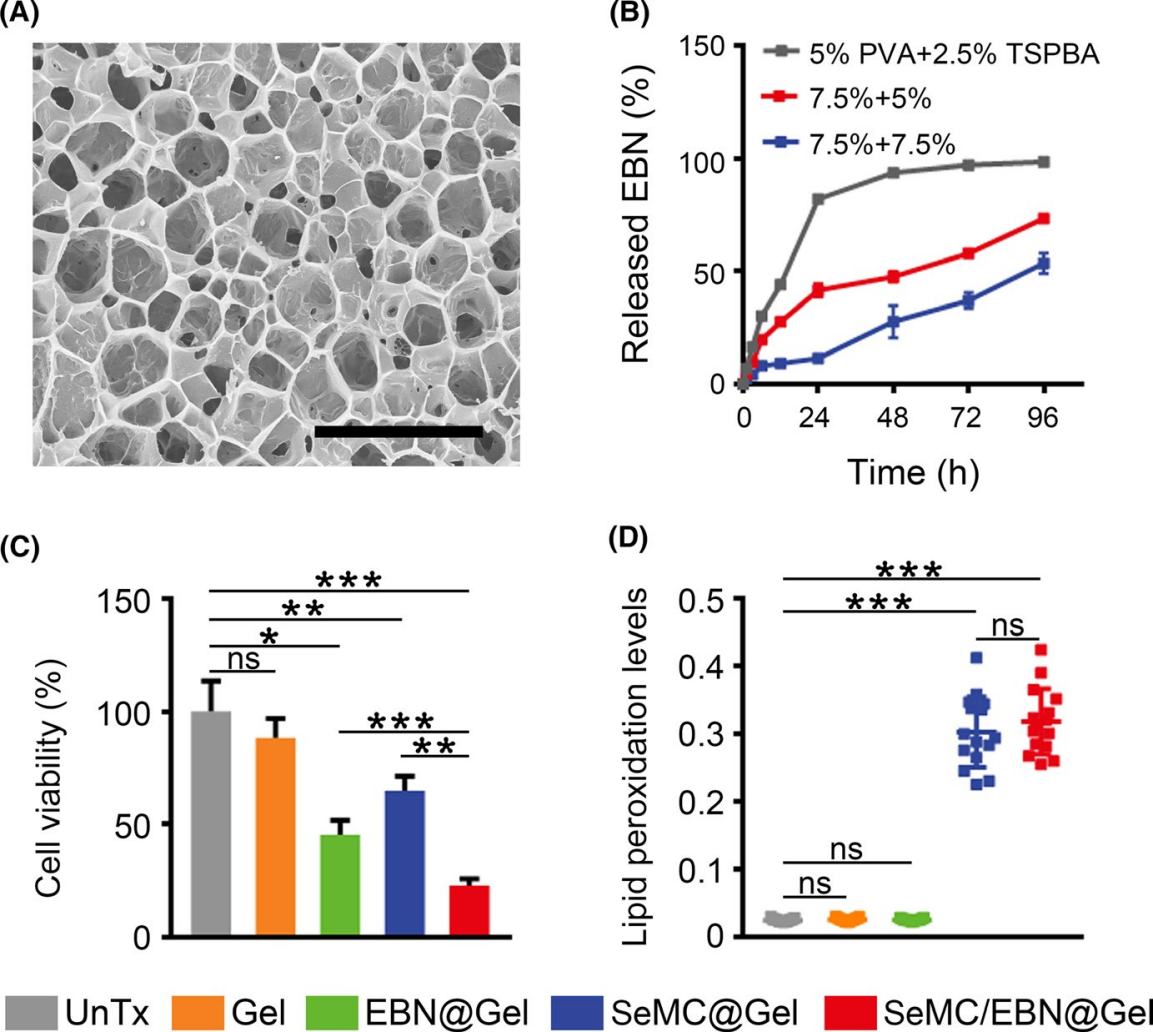
Characterization of drug‐loaded hydrogel. (A) Cryo‐SEM imaging of the hydrogel composed of 7.5 wt% PVA matrix and 5 wt% TSPBA linker. Scale bar: 5 μm. (B) Disassembly kinetics of EBN@Gel with different PVA and TSPBA concentrations in the presence of 0.5 mM H_2_O_2_. Data are represented as mean ±SD (n = 3). (C) Viabilities of A549 cells incubated with the supernatant from Gel, SeMC@Gel, EBN@Gel or SeMC/EBN@Gel. The concentrations of SeMC and EBN in the supernatant were 200 μM and 0.2 μg/mL, respectively. Data are represented as mean ±SD (n = 3). (D) Relative lipid peroxidation levels in A549 cells treated with the supernatant from Gel, SeMC@Gel, EBN@Gel or SeMC/EBN@Gel are represented as the ratio of oxidized to reduce BODIPY‐C11. The concentrations of SeMC and EBN in the supernatant were 200 μM and 0.2 μg/mL, respectively. Data are represented as mean ±SD (n = 15). *P < .05, **P < .01, ***P < .001, ns means not significant

Since both SeMC and EBN could increase intracellular oxidative stress, it would be ideal to restrict their activities mainly in the tumour tissue. Therefore, we resorted to the hydrogel drug delivery platform for local combination therapy. We utilized a type of ROS‐responsive hydrogel composed of the PVA matrix and the TSPBA linker. The two components could quickly form the hydrogel network in situ upon mix and sensed high levels of ROS in the TME for sustained hydrogel disassembly and drug release. The hydrogel scaffold exhibited porous structure in the cryo‐scanning electron microscopy (Cryo‐SEM) for efficient drug loading (Figure 4A). Subsequently, we employed the EBN‐loaded hydrogel (EBN@Gel) to evaluate the degradation dynamics and drug release profile of the hydrogel in the presence of ROS stimuli. The rate of EBN release depended on the concentration of the PVA matrix and that of the TSPBA linker. Notably, hydrogel composed of 7.5 wt% PVA and 5 wt% TSPBA displayed a moderate disassembly rate that was appropriate for in vivo application, and around 70% of the EBN was released in four days (Figure 4B). We assessed the efficacy of drug‐loaded hydrogel in cultured A549 cells by adding the supernatant from degraded hydrogel into the cell media. Unloaded hydrogel (Gel) did not affect the viability of A549 cells. EBN@Gel and SeMC‐loaded hydrogel (SeMC@Gel) showed moderate inhibitory effects, and the hydrogel loaded with both drugs (SeMC/EBN@Gel) exhibited the strongest effect on cell viability (Figure 4C). Moreover, we measured the capacity of drug‐loaded hydrogel in the induction of lipid peroxidation. Incubation with the supernatant from control Gel or EBN@Gel did not trigger lipid peroxidation. SeMC@Gel and SeMC/EBN@Gel exhibited comparable ability in the generation of lipid peroxides, validating the effectiveness of SeMC release from the hydrogel (Figure 4D and Figure S4). Collectively, above results confirmed the feasibility of the hydrogel platform as a carrier for spatiotemporally controllable administration of therapeutics.

### 3.5 Hydrogel‐mediated drug delivery enhances anti‐tumour activity

**FIGURE 5:**
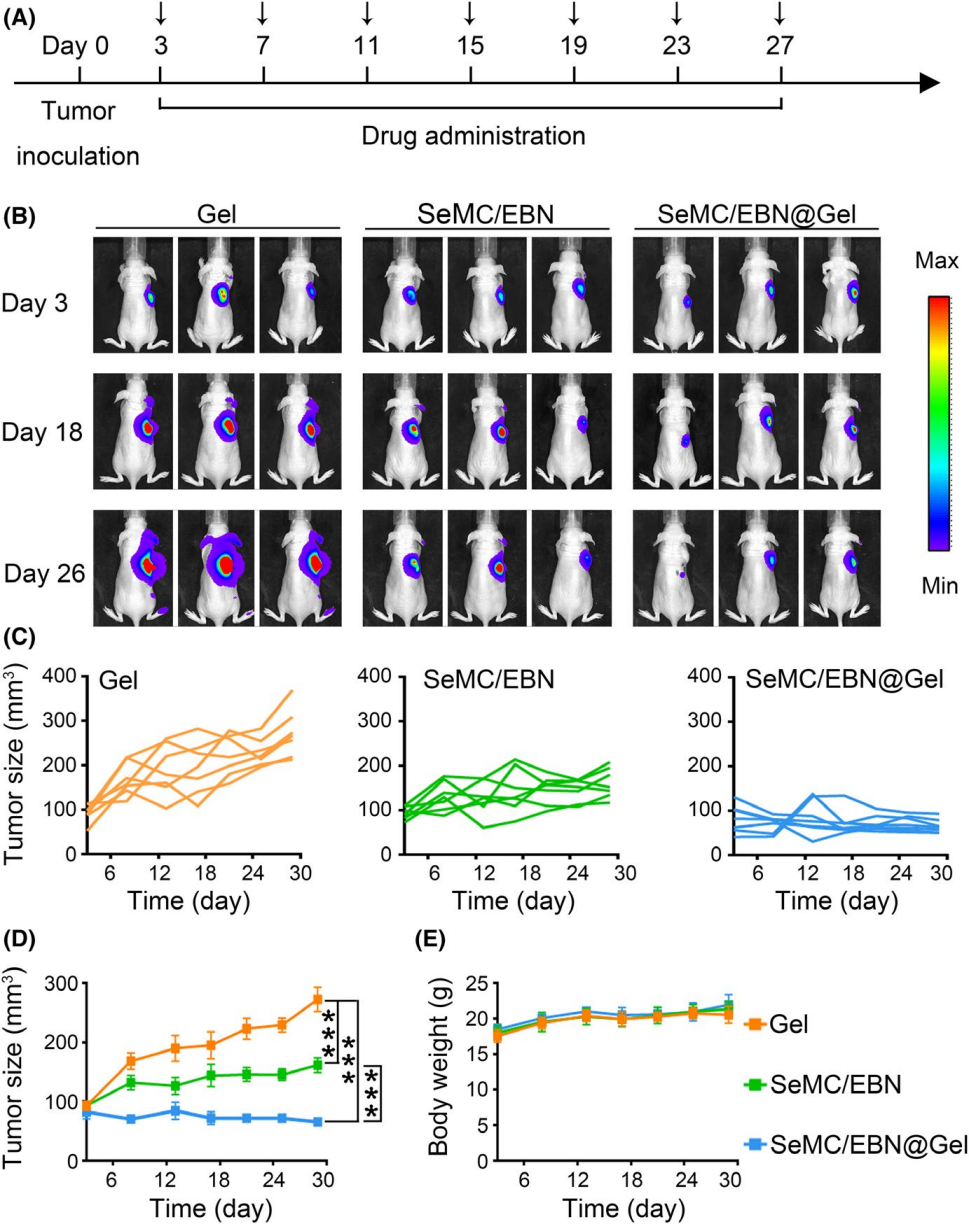
Drug‐loaded hydrogel enhances tumour suppression. (A) Schematic illustration of drug administration in tumour‐bearing mice. (B) Representative bioluminescence imaging for indicated groups. (C) Individual tumour growth kinetics (n = 7). (D) Average tumour growth curves for indicated groups. Data are represented as mean ±SEM (n = 7). ***P < .001. (E) Body weight of tumour‐bearing mice in indicated groups. Data are represented as mean ±SD (n = 7)

Having established the efficiency of drug‐loaded hydrogel in cultured cells, we continued to explore its efficacy in mice bearing A549 carcinomas. We intratumorally injected SeMC/EBN@Gel every four days (from day 3 to day 27). As comparison, the control Gel and free SeMC/EBN were injected with the same interval (Figure 5A). Bioluminescence signal from the A549 cells indicated that repeated administration with free SeMC/EBN moderately inhibited tumour progression, whereas the SeMC/EBN@Gel dramatically suppressed tumour growth (Figure 5B). These results were confirmed by the measurement of tumour sizes (Figure 5C,D). Comparing to the mice treated with unloaded hydrogel, which exhibited rapid tumour progression, administration of free SeMC/EBN reduced the mean tumour volume by 40.7 ± 12.3%. Hydrogel‐mediated delivery of SeMC/EBN significantly enhanced their inhibitory effects on tumour growth and reduced the mean tumour volume by 76.0 ± 5.7%. Meanwhile, we observed no significant changes in the body weight among different groups, suggesting that our therapeutic strategies did not trigger severe side effects (Figure 5E). Together, these results supported our hypothesis that the in situ formed hydrogel could enhance the anti‐tumour efficacy of SeMC and EBN combination therapy.

## 4 DISCUSSION

The comprehensive effects of organic Se compounds on cancer cell proliferation are controversial.^31^, ^32^, ^33^ Here, we monitored the viabilities of a series of different cell lines in the presence of SeMC. At concentrations between 50 and 200 μM, most tested cell lines showed good tolerance to SeMC exposure. However, we also found that A549 cells are more susceptible to SeMC treatment than other types of cells including 293T, L6, 4T1, CT26 and Hepa1‐6 cells. While these results suggest that the effects of SeMC can be cell‐specific, the precise mechanism underlying these differences are not clear. Given SeMC needs to be metabolized before cellular utilization,^34^, ^35^, ^36^ one possible explanation is that different types of cells accumulate harmful metabolites of SeMC at different rates.

The inhibitory effect of SeMC on A549 cells prompted us to test the efficacy of its combination with chemotherapeutics. We found that SeMC showed a broad spectrum of augmentation on the efficacy of various types of chemotherapeutic agents. Therefore, the combination of SeMC with chemotherapeutics could reduce the required doses of drugs for cancer therapy and avoid potential side effects. We further found that SeMC‐induced the generation of lipid peroxides, which might sensitize the A549 cells to chemotherapeutics.

Hydrogel‐based drug delivery platforms are promising tools to provide sustained drug release, increase drug concentration at tumour foci and reduce systemic toxicity.^24^ Considering the abundant ROS in the TME,^37^, ^38^ we constructed a hydrogel scaffold that was responsive to ROS for local SeMC and EBN combination therapy. As expected, treatment with SeMC/EBN@Gel inhibited tumour progression more efficiently than the free drugs. In our experimental design, SeMC/EBN@Gel was administrated every four days. We expect that the interval of administration can be further prolonged via the improvement of the hydrogel network.

In conclusion, our findings suggest cell‐specific effect of SeMC on different types of cancer cells. Our data indicate that SeMC induces lipid peroxidation to increase ROS generation, and thereby potentiates the efficacy of therapeutic agents. We show the potential of SeMC in tumour inhibition via combined treatment with chemotherapeutics and develop a hydrogel‐based drug delivery strategy that achieves much higher efficiency than conventional drug administration.

## CONFLICT OF INTEREST

All authors of this paper declare no conflict of interest.

## AUTHOR CONTRIBUTIONS

HS and JZ designed and supervised the research; JM and JH performed the experiments and wrote the manuscript; JS, DG, CL and JL assisted the experiments in cultured cells; YZ and XJ assisted animal treatment and data analysis.

## Supporting information

Figure S1‐S4

## Data Availability

The data supporting the findings of this study are available from the corresponding authors upon reasonable request.
