# Supplementary material for: L‐Se‐methylselenocysteine sensitizes lung carcinoma to chemotherapy
Source: Cell Prolif. 2021 Apr 1;54(5):e13038. doi: 10.1111/cpr.13038 (PMC8088472; doi:10.1111/cpr.13038)
Supplement: Supplementary file 1 — Figure S1‐S4 [file CPR-54-e13038-s001.docx]

**Supporting Information**

**L-Se-Methylselenocysteine Sensitizes Lung Carcinoma to Chemotherapy**

Jia Ma,^1,2,‡^ Jing Huang,^3,‡^ Jinli Sun,^2^ Yanfeng Zhou,^2^ Xiaoyuan Ji,^2^ Daoxia Guo,^2^ Chang Liu,^2^ Jiyu Li,^4^ Jiye Zhang,^1,*^ Haiyun Song^2,*^

^1^School of Pharmacy, Health Science Center, Xi’an Jiaotong University, Xi’an 710061, China

^2^State Key Laboratory of Oncogenes and Related Genes, Center for Single-Cell Omics, School of Public Health, Shanghai Jiao Tong University School of Medicine, Shanghai 200025, China

^3^Department of Neurology, Xuhui District Central Hospital, Shanghai 200032, China

^4^Henan Xibaikang Health Industry Co., Ltd., Jiyuan 459000, China

^*^Correspondence: Jiye Zhang, zjy2011@mail.xjtu.edu.cn;

Haiyun Song, songhaiyun@shsmu.edu.cn

^‡^These authors contributed equally to this work.


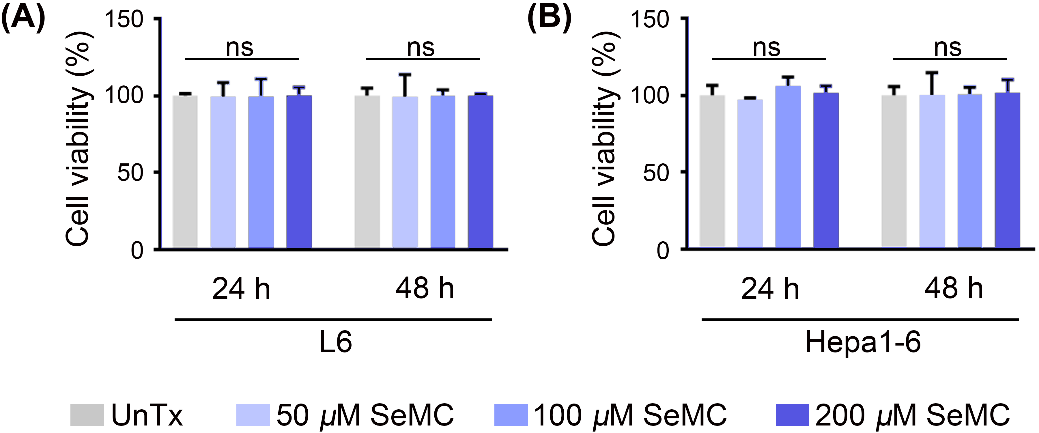


**Figure S1 Biocompatibility of SeMC in L6 cells and Hepa1-6 cells.** (A-B) Cytotoxicity assays in L6 cells (A) and Hepa1-6 cells (B) in the presence of SeMC for 24 hours or 48 hours. Data are represented as mean ± SD (n=3). ns means not significant.


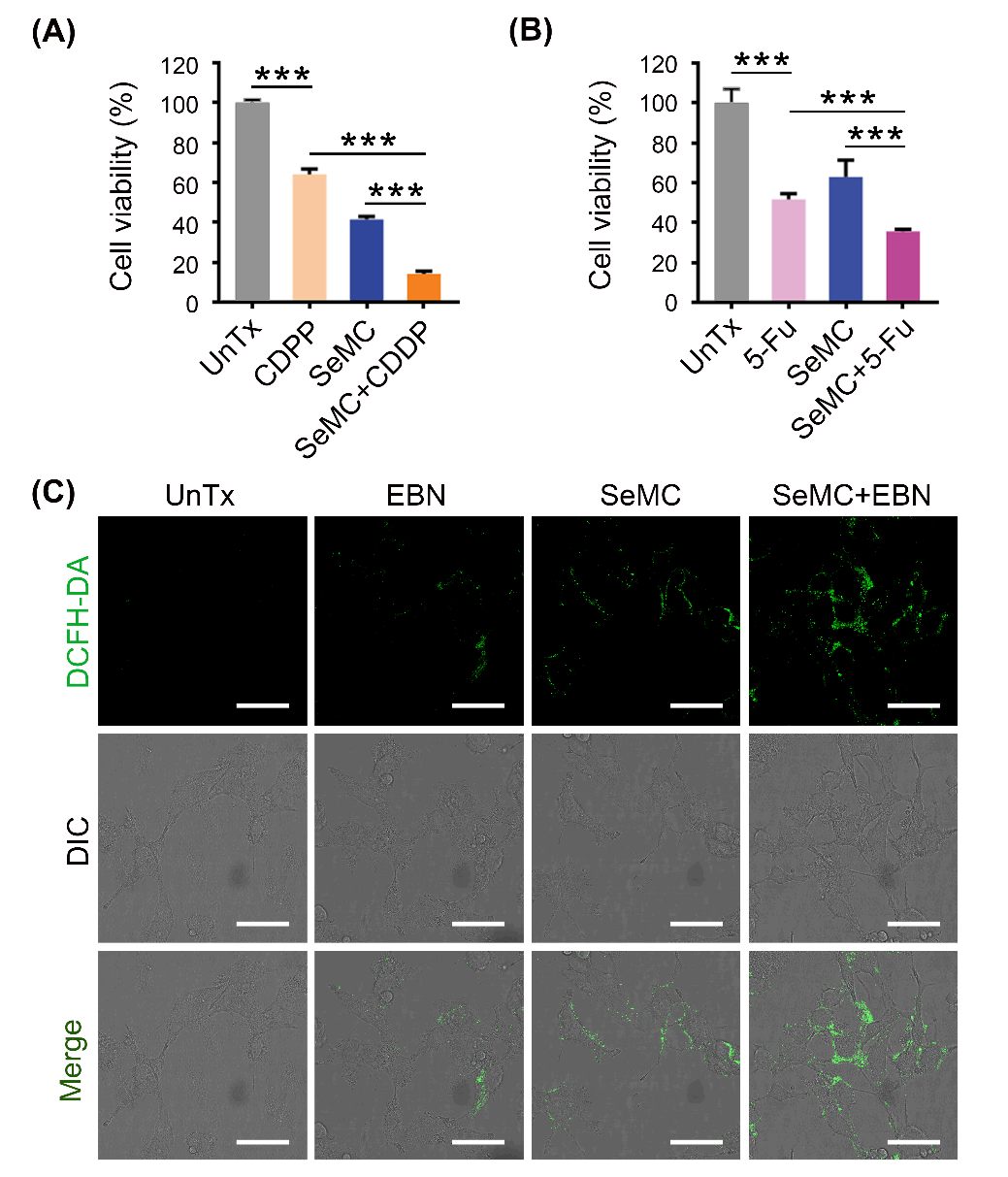


**Figure S2 SeMC acts synergistically with chemotherapeutic agents.** (A-B) The viabilities of A549 cells after exposure to 200 *μ*M SeMC, 3 *μ*g/mL CDDP, 2.5 *μ*g/mL 5-Fu or their combination for 48 hours. Data are represented as mean ± SD (n=3). *** *P* < 0.001. (C) Fluorescent images of ROS levels in A549 cells after indicated treatments. Scale bars: 50 *μ*m.


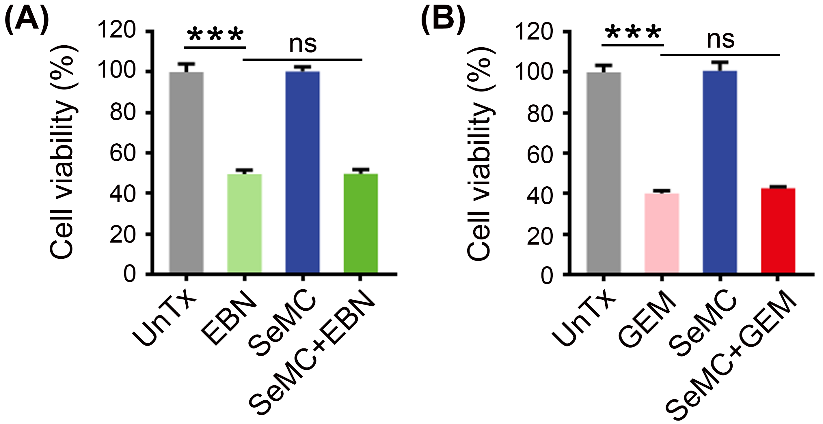


**Figure S3 SeMC does not sensitize 4T1 cells to chemotherapeutic agents.** (A-B) The viabilities of 4T1 cells after exposure to 200 *μ*M SeMC, 0.2 *μ*g/mL EBN, 0.5 *μ*g/mL GEM or their combination for 48 hours. Data are expressed as mean ± SD (n=3). *** *P* < 0.001, ns means not significant.

**
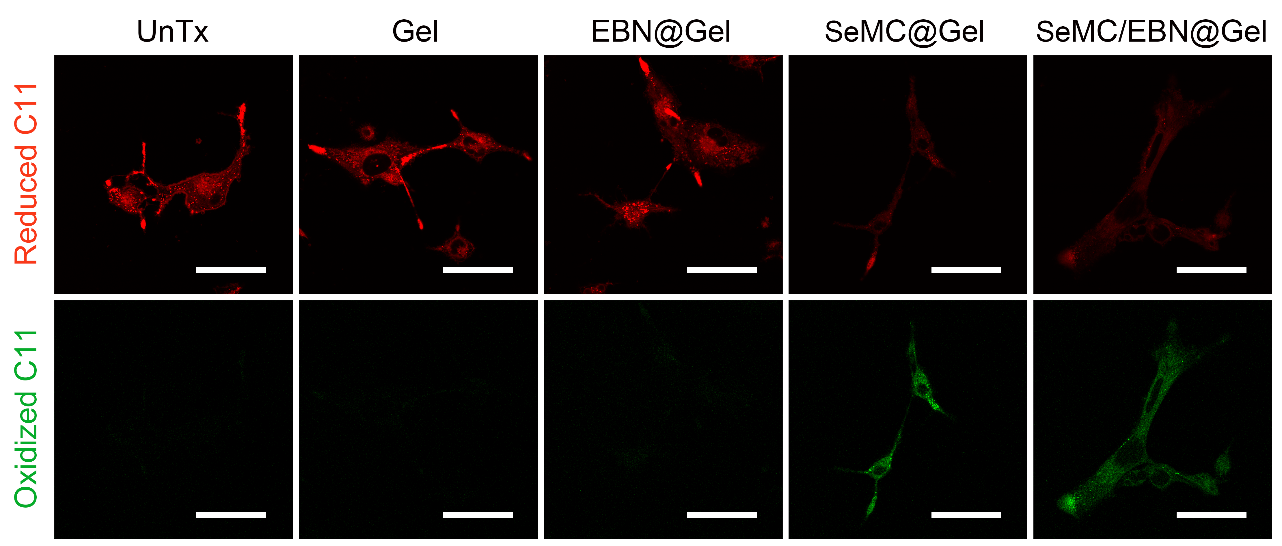
**

**Figure S4 SeMC-loaded hydrogel induces lipid peroxidation.** Confocal images showing reduced form (red) and oxidized form (green) of BODIPY C11 in A549 cells after incubation with the supernatant from Gel, SeMC@Gel, EBN@Gel or SeMC/EBN@Gel. The concentrations of SeMC and EBN in the supernatant were 200 *μ*M and 0.2 *μ*g/mL, respectively. Scale bars: 50 *μ*m.
